# Regulatory T Cells Beyond Autoimmunity: From Pregnancy to Cancer and Cardiovascular Disease

**DOI:** 10.3389/fimmu.2020.00509

**Published:** 2020-03-31

**Authors:** Elisa Martini, Silvia Giugliano, Maria Rescigno, Marinos Kallikourdis

**Affiliations:** ^1^Adaptive Immunity Laboratory, Humanitas Clinical and Research Center, Milan, Italy; ^2^Laboratory of Mucosal Immunology and Microbiota, Humanitas Clinical and Research Center, Milan, Italy; ^3^Department of Biomedical Sciences, Humanitas University, Milan, Italy

**Keywords:** Treg, pregnancy, autoimmunity, cancer, cardiovascular disease, microbiota, evolution

## Abstract

The evolution of the full range of functions of regulatory T cells (Treg) coincides with the evolution of mammalian pregnancy. Accordingly, Treg function has been shown to be crucial for maternal-fetal tolerance and implantation. As reproduction is a key point of selective pressure, mammalian pregnancy may represent an evolutionary driver for the development of Treg. Yet beyond the chronological boundaries of mammalian pregnancy, several key physiological and pathological events are being gradually uncovered as involving the immunomodulating functions of Treg cells. These include autoimmunity, age-related inflammation in males and in post-menopausal females, but also oncological and cardiovascular diseases. The latter two sets of diseases collectively compose the main causes of mortality world-wide. Emerging data point to Treg-modulable effects in these diseases, in a departure from the relatively narrower perceived role of Treg as master regulators of autoimmunity. Yet recent evidence also suggests that changes in intestinal microbiota can affect the above pathological conditions. This is likely due to the finding that, whilst the presence and maintenance of intestinal microbiota requires active immune tolerance, mediated by Treg, the existence of microbiota *per se* profoundly affects the polarization, stability, and balance of pro- and anti-inflammatory T cell populations, including Treg and induced Treg cells. The study of these “novel,” but possibly highly relevant from an ontogenesis perspective, facets of Treg function may hold great potential for our understanding of the mechanisms underlying human disease.

## Introduction: Selective Pressure Shapes Function in Treg

Biological systems develop as serendipitous solutions to selective pressure, according to evolutionary theory. The evolution of regulatory T (Treg) cells and their master regulator transcription factor, *foxp3* (1), must have occurred in response to selective pressure that conferred an advantage to vertebrates that possessed them. Whilst an early form of *foxp3* does exist in zebrafish (2), the full set of domains of *foxp3* only appear in the non-placental mammal platypus (3). Additionally, the enhancer element that is necessary for the induction of induced Treg (iTreg) in the periphery, also first appears in the platypus (4).

The platypus is an egg-laying mammal, and the egg creates a barrier separating the (non-self) paternal antigens from the maternal adaptive immune system. Absence of a barrier would necessitate a mechanism of suppression of maternal anti-fetal responses, a requirement termed “immunological paradox of pregnancy” by transplantation pioneer Medawar (5). On the other hand, all subsequent (in terms of speciation) mammals are placental, having dispensed with the egg, benefiting from the advantage of a continuous flow of nutrients to the fetus. Thus, one can speculate that the serendipitous acquisition of an immunosuppressive T cell subpopulation could have enabled the elimination of the egg barrier.

In support of such a speculation we and others have shown that placental pregnancy with a genetically different father is not possible in the absence of regulatory T cells (6–8). Defects in Treg cells are associated with increased early-stage miscarriage and preeclampsia in humans (9, 10). In summary, whilst a robust adaptive immune system, as developed in vertebrates, is essential in maintaining defense of the self against pathogens (11), the evolution of Treg cells in placental mammals may have enabled the more complex management of the distinction between the self vs. the “non-self of the same species”. The recognition of non-self of the same species, which is central in placental pregnancy, is ironically a much older problem, as sea-dwelling protochordate *Botryllus* had to fend off -and not tolerate- competition from neighboring individuals of the same species, using molecular processes not too dissimilar from those of Natural Killer (NK) cells (12). In mammalian pregnancy, maternal uterine NK cells interacting with non-classical Class I Major Histocompatibility molecules, such as HLA-G, independent of presence or absence of alloantigen, are essential for vascularization of the placenta, especially at the start of pregnancy (13, 14).

## Treg in Pregnancy: A Fluctuating But Regulated Population

Evidence from mice and humans demonstrates that the abundance of Treg cells is modified during events linked to placental pregnancy. Periodic fluctuations in uterine (15) or peripheral (16) Treg levels render the cells more abundant during the fertile window of the estrus/menstrual cycle, so that suppression can take place should a pregnancy occur. These fluctuations are possibly estrogen-driven, as estrogen has been shown to boost Treg function (17, 18), whilst estrogen-depleting ovariectomy reduces Treg cell abundance (19). Once fertilization occurs, a much more substantial expansion of Treg cells can be observed (6). In this expansion, a role for paternal and male antigen-driven expansion of Treg has been demonstrated; initially in response to seminal fluid antigens (20), as well as paternal antigens (8, 21), which may explain the clonal expansion of Treg cells in the decidua but not the periphery in pregnant women (10).

Intriguingly, the pregnancy-associated expansion can be interrupted, should a uterine infection appear that could jeopardize fetus and mother (22). From a speculative evolutionary perspective, pathogen-induced reductions in Treg functionality would be selected for, as they would spare the mother from pathogens that could expand uncontrollably in an immunosuppressed environment. A putative mechanism may involve recognition of the pathogen by IL-6-producing innate immune cells, blocking the suppressive potential of Treg (23). Indeed, IL-6 is associated with fertility and pregnancy-related pathologies (24), and the cytokine is also known to mediate a conversion of Treg cells into Th17 pro-inflammatory cells in autoimmune arthritis (25). It should be noted that danger signal-induced fetal rejection can be mediated by invariant/semi-invariant lymphocytes, such as iNKT cells (26), Mucosal-Associated Invariant T cells (27) or γδ T cells (28).

## Treg in Autoimmunity

It is reasonable to ask how the function most often ascribed to Treg cells, the control of autoimmunity, fits with their role in placental reproduction. Pregnancy is known to temporarily alleviate the symptoms of rheumatoid arthritis in a majority of patients (29). In a murine model of autoimmune arthritis we have shown that the pregnancy-driven expansion of Treg is indeed responsible for this amelioration (30).

**Figure 1:**
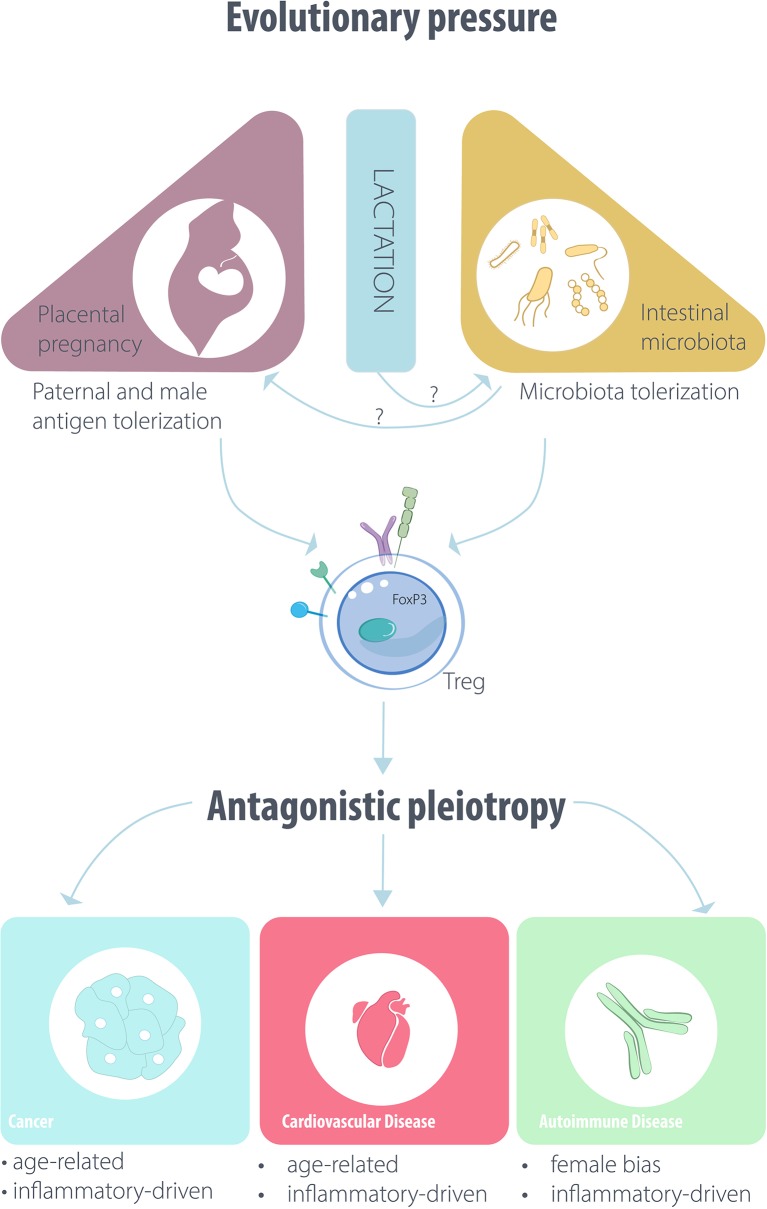
Outline of the putative evolutionary drivers affecting Treg/iTreg function and disease pathogenesis.

A few studies on peripheral blood Treg levels in human patients have shown a maintenance of their elevated numbers in the short period post-partum (31). Nonetheless, in antigen-specific murine models, which can be monitored with more precision, long-term increases in Treg levels from previous pregnancies were much lower than the peak reached during pregnancy (8). Consequently, Treg levels must be dropping after every pregnancy. The same could occur at menopause, as the ovariectomy-induced Treg contraction suggests (19). The time period post-partum and menopause are the main windows of incidence of most -though not all (32)—autoimmune diseases, which often affect far more women than men. In a logical corollary of an evolutionary selection-driven role for Treg in pregnancy, this higher incidence of autoimmunity in women may be an unwanted consequence of an estrogen-responsive Treg population that is necessary for an improved, placental reproduction. Such unwanted deleterious effects of selected-for earlier benefits fall within the term antagonistic pleiotropy (Figure 1), often applied to describe the benefit of infection-fighting immunity in young age leading to deleterious inflammation in old age (33). As pregnancy is the timepoint of genetic heredity, conditions after pregnancy may potentially feature antagonistic pleiotropy effects.

## After Menopause and in Men: Age-Related Diseases

The absence of estrogen fluctuation in ovariectomized female mice induces not only a reduction in Treg levels but also a shortening of their lifespan, which approaches that of male mice (19). This is compatible with an overall beneficial effect of Treg in females undergoing estrus/menstrual cycles. If this conjecture were to be true, it would lead to a propensity for inflammation-associated disease in post-menopause women and in all men. Murine models may not be the optimal subjects of study for this question, as mice do not undergo menopause; only humans and cetaceans do (34), possibly as a result of socially-driven selection. Further, modern humans have another unique feature: compared to other primates or even human hunter-gatherer populations, modern humans live substantially longer, with longevity data forming a branching point corresponding to the first industrialized societies (circa 1850), when sanitation was applied in a large scale (35). We can therefore hypothesize that if Treg function has been selected, in all mammals from the platypus onwards, thanks to its beneficial effects in enabling pregnancy, it would still nonetheless be involved, beneficially or detrimentally, in the regulation of any inflammation-associated ailment that occurs in our recently-acquired long lifespans. The reported decay of thymic Treg and iTreg with advanced age (36) could indeed contribute to a loss of self-tolerance to antigens expressed by aging tissue, promoting inflammation-associated disease pathogenesis. The ailments that predominantly affect older humans and lead to the majority of human mortality in the developed world are cancer and cardiovascular disease (37). Fitting our conjecture above on a Treg-related benefit prior to menopause, cardiovascular disease has higher incidence in men than in women, with a difference that decreases with older age (38). For all these reasons, thus, we would expect to find regulatory roles for Treg cells within these groups of diseases.

## Treg and Cancer

The link between inflammation and cancer is two-pronged. On the one hand, extensive findings have demonstrated that pro-inflammatory cytokines may enhance the chances of carcinogenesis and genetic instability (39). Treg-mediated suppression of such oncogenic inflammation would be beneficial. Such events clearly happen away from the clinically observable conditions of cancer patients, whose diagnosis occurs long after the carcinogenic event; this may be limiting the incentive to study the role of Treg cells in carcinogenesis. Yet even in growing tumors, evidence has shown that formation of tumor-promoting fibrotic capsules around prostate tumors occurs only in the presence of pro-inflammatory T cells (40), selective suppression of which would be beneficial.

On the other hand, the most clinically important interaction between immunity and cancer is the anti-tumoral, pro-inflammatory function of immunosurveillance (41), which has enabled the development of tumor immunotherapy. The latter, in its most applicable form of immune checkpoint blockade immunotherapy, is based on antibody-mediated reactivation of pro-inflammatory T cells. Yet Treg cells express and utilize the immunotherapy target molecules CTLA-4 (42) and PD-1 (43), and the suppressive action of the Treg may be inhibiting beneficial anti-tumor immunity (44). Why would Treg cells inhibit an anti-tumor response? Interpreted according to the signals a Treg cell may have evolved to deal with, a tumor expressing self-antigens and neo-antigens may be not that different from a fetus, the putative driver of the Treg cells' selection. Genes and processes that help fight non-pediatric, growing, solid tumors cannot have been inherited and selected for in mammals, as until very recently it was not possible to survive and reproduce following cancer incidence.

And yet an obvious solution does arise from the, admittedly speculative, study of the evolutionary drivers of Treg function. As hypothesized above, Treg suppression could collapse in order to reject an infected fetus, in order to protect the mother from the infecting pathogen. In this context, as pioneered in principle by Coley's toxin (45), vaccination strategies that fool the immune system into identifying the tumor as an infected fetus may represent tools that are aligned with the evolutionary drivers of the biological components that we are trying to modulate (46).

## Treg and Cardiovascular Disease

Cardiovascular diseases, ranging from atherosclerosis to myocardial infarction (MI) and heart failure, are not traditionally thought of as linked to immunity. Over the recent years, experiments showing that stressed cardiomyocytes release pro-inflammatory cytokines (47) led to clinical trials aiming to therapeutically inhibit the cytokine activity via monoclonal antibodies. As these failed (48), renewed efforts centered on identifying the adaptive immune cells involved in the progression of pathogenesis in atherosclerosis and heart failure. This was based on the premise that a chronic immune response may well be under the control of adaptive immunity. Accordingly, a role for Treg was identified in atherosclerosis (49), whilst therapeutic effects by the experimental administration of Treg cells in a model of pressure overload induced heart failure were also reported (50). In a more translational approach, we have used a molecule derived from Treg, CTLA-4, in soluble fusion protein form (CTLA-4-Ig/Abatacept) to treat advanced-stage heart failure in the pressure overload model. Surprisingly, the treatment with the drug, which is FDA-approved for use in Rheumatoid Arthritis patients, was almost 3-fold more effective than the current standard therapy for heart failure, demonstrating the potential of Treg-inspired therapeutic strategies (51).

More recently we identified, via single cell RNA sequencing, that Treg cells found to be infiltrating the ailing myocardium, express PD-1. Inhibition of PD-1 in healthy hearts blocked the Treg-mediated suppression, releasing cardiac inflammation, which in turn led to a significant reduction in heart function (52). This is intriguing, as anti-PD-1 treatment in human cancer patients has been shown to occasionally lead to T cell-mediated fulminant myocarditis (53). Luckily a solution exists, as CTLA-4-Ig treatment of tumor immunotherapy-induced myocarditis patients has a rescuing effect (54).

In MI that progresses to chronic ischemic heart failure, very recent evidence suggests that Treg cells may lose their immunosuppressive properties, becoming pro-inflammatory and worsening disease outcome (55). Their role is somewhat less clear in the early phase of post-MI repair, where the pro-inflammatory conventional T cells may be useful in the short term in order to deal with the extensive tissue damage (56, 57).

## Treg and Infections

Treg can dampen the response against pathogens during an infection, limiting collateral damage. As a consequence, this also leads to pathogen persistence, which in turn boosts the persistence of protective immunity against the pathogen itself (58). Yet, simultaneously, the inflammation associated with the response limits the functionality of Treg cells (59), a finding that matches the inhibition of Treg function induced by danger signals, mentioned above (23), or indeed by inflammation *per se*, including in contexts of cardiovascular disease (60).

## Treg and Intestinal Microbiota

The fetus expressing paternal antigens is not the only “non-self” that our adaptive immune system has to tolerate via Treg cells. Intestinal microbiota are essential for our survival and are not rejected (61), despite reaching very high cell numbers in the gut (62). The tolerization of “useful” bacteria may be mediated via Treg-mediated suppression (63), whereas “harmful” bacteria may be attacked by pro-inflammatory T cell subpopulations (64). Conversely, both anti-inflammatory iTreg and pro-inflammatory Th17 cells are induced in the gut, displaying a plasticity that depends on the microbiota (65, 66). For example, the immunomodulatory capsule polyaccharide A (PSA) of *Bacteroidetes fragilis* has been shown to induce IL-10-secreting Treg cells in the gut, restraining gut inflammation (67). Further, bacterial metabolites such as short-chain fatty acids (SCFAs), are involved in Treg differentiation (68–70).

Consequences of the microbiota-induced plasticity may affect disease pathogenesis. The anti-tumoral, pro-inflammatory effect of anti-CTLA-4 or anti-PD-1, described above, was abolished in experimental systems where the intestinal microbiota were eliminated (71, 72), demonstrating the potency of the microbiota-mediated effects. In agreement with these striking results, multiple translational studies have now highlighted how the microbiome of patients that respond to anti-PD-1 treatment is significantly different from that of non-responders (73), and how antibiotic treatment in combination with anti-PD-1/anti-PD-L1 immunotherapy can have a direct effect on patient survival rate (74).

In an analogous manner, emerging evidence demonstrates that the microbiome can significantly affect the pathogenesis and outcome of cardiovascular disease. Alteration of the gut microbiota has been associated with atherosclerotic lesion formation, as revealed by gut metagenome analysis in patients (75). Gut microbiota-produced SCFAs have even been shown to affect blood pressure regulation (76). The above findings exemplify how gut microbiota, possibly also via their effects on iTreg/Th17 populations, have substantial, though still largely unexplored, regulatory roles on the major disease groups that drive mortality world-wide.

## Concluding Remarks—An Evolutionary Role for Mammalian Gut Flora Upstream of Treg and Placental Pregnancy?

The hypothesis that pregnancy may have been a driver for the selection and survival of estrogen-responsive, immunosuppressive Treg cells that enable us to reproduce via a placenta and not using eggs is attractive. The enhanced ability to provide a continuous flow of nutrients to the fetus could even be hypothesized to have helped select bigger brains, capable of abstract thought. However, the interaction between Treg cells and the intestinal microbiota, which is central to our physiology and pathology, raises an intriguing possibility. The evolution of fully-functional *foxp3* may have indeed been a prerequisite for the evolution of placental mammalian pregnancy. At least until further species are sequenced, the full set of *foxp3* domains appears to have first appeared in the platypus. Yet Treg evolution in the platypus cannot have had pregnancy as a driver of selective pressure, as the platypus is lactating but does not have a placenta. We could, however, hypothesize that, prior to pregnancy, the intestinal microbiota required to digest milk in mammals, which first appeared in the platypus, offering clear advantages as a source of readily-available calories for the litter, could be distinctly different from those found in non-mammalian vertebrates. Could such a bacterial diversification drive the selection of a dedicated tolerizing immune cell population? Could this immunosuppressive population only then enable the evolution of pregnancy in the next speciation step? Compatible with this hypothetical conjecture, the intestinal microbiota of lactating animals has been recently found to be significantly different (77). Further experimentation will be required to assess the validity of these hypotheses.

The interaction between intestinal microbiota and Treg, especially if the conjecture that the former may have been a driver for the evolution of the latter is valid, offers novel means of investigating the functional aspects of Treg cells. In the long term, one would hope that this will lead to innovative therapeutic strategies, in the context of autoimmunity, cancer and cardiovascular disease.

## Author Contributions

All authors listed have made a substantial, direct and intellectual contribution to the work, and approved it for publication.

### Conflict of Interest

The authors declare that the research was conducted in the absence of any commercial or financial relationships that could be construed as a potential conflict of interest.
